# Physical exercise during adjuvant chemotherapy for colorectal cancer—a non-randomized feasibility study

**DOI:** 10.1007/s00520-020-05789-z

**Published:** 2020-10-08

**Authors:** I. Hatlevoll, L. M. Oldervoll, A. Wibe, G. B. Stene, S. N. Stafne, E. Hofsli

**Affiliations:** 1 Department of Oncology, St. Olav’s Hospital, Trondheim University Hospital, Trondheim, Norway; 2 Department of Cancer Research and Molecular Medicine, Norwegian University of Science and Technology, Trondheim, Norway; 3 Department of Public Health and Nursing, Norwegian University of Science and Technology, Trondheim, Norway; 4 LHL-clinics, Trondheim, Norway; 5 Department of surgery, St. Olav’s Hospital, St. Olav’s Hospital, Trondheim, Norway; 6 Department of Neuromedicine and Human Movement Science, The Faculty of Medicine and Health, Norwegian University of Science and Technology, Trondheim, Norway; 7 Department of Clinical Services, St. Olav’s Hospital, Trondheim University Hospital, Trondheim, Norway

**Keywords:** Colorectal cancer, Physical exercise, Adjuvant chemotherapy, Neuropathy, Oxaliplatin

## Abstract

**Background:**

Colorectal cancer (CRC) is the third most common cancer worldwide, and a large proportion of the patients receive adjuvant oxaliplatin-based chemotherapy. Most of these experience chemotherapy-induced peripheral neuropathy (CIPN), affecting quality of life. Evidence to advise exercise to reduce CIPN is limited. The primary aim of this study was to investigate the feasibility of an exercise intervention and data collection among CRC patients during adjuvant chemotherapy.

**Material and methods:**

This non-randomized feasibility study included CRC patients admitted to adjuvant chemotherapy to an intervention consisting of supervised aerobic endurance, resistance, and balance exercises twice a week at the hospital in addition to home-based exercise once a week. A physiotherapist supervised the patients, and the intervention lasted throughout the period of adjuvant chemotherapy (12–24 weeks). Participants performed physical tests and filled in questionnaires at baseline, 3, 6, 9, and 12 months.

**Results and conclusion:**

Nineteen (63%) of 30 invited patients consented. A major barrier to recruit or consent to participation was long travel distance to the hospital. The completion rate of questionnaires and physical tests were near 100%. Seven participants dropped out, five before the intervention started. Median attendance to supervised exercise was 85%. There were no serious adverse events related to the intervention. Except for a planned higher intensity of endurance exercise, we found the intervention feasible and safe. Based on experiences in this study, some adjustments have been made for an upcoming randomized trial, including the supervised exercise taking place close to participants’ homes.

**Trial registration:**

NCT03885817, March 22, 2019, retrospectively registered.

## Introduction

Colorectal cancer (CRC) is the third most common cancer worldwide with 1.8 million new cases each year [1]. Adjuvant chemotherapy is a standard treatment for stage III and some high-risk stage II colon cancer [2]. In addition, postoperative chemotherapy is considered after surgery for stage IV CRC and after resection of locally advanced rectal cancer. Chemotherapy can cause several short- and long-term side effects, which may have major negative impacts on patients’ quality of life [3–5]. Chemotherapy-induced peripheral neuropathy (CIPN) is a frequent side effect from oxaliplatin, which is used in the adjuvant treatment of CRC, with more than 90% of the patients exposed to the compound experiencing CIPN [6].

According to recent guidelines, there is strong evidence to advise cancer patients to carry out aerobic exercise alone or in combination with resistance training at moderate intensity, both during and after treatment, to improve several cancer-related health outcomes [7]. Also, there are exercise guidelines for cancer survivors based on guidelines for the general population with both moderate-intensity and vigorous physical activities [8]. Notably, current recommendations are mainly based on evidence from clinical trials conducted in breast or prostate cancer patients. Less is known about the effects of higher-intensity aerobic exercise during cancer treatment, and studies on this topic are scarce. Independent of outcomes, few randomized controlled trials (RCTs) have investigated the effects of exercise during adjuvant chemotherapy among CRC patients, and in available studies, the sample sizes are small [9]. To our knowledge, there are no trials exploring the effects of a combination of supervised and home-based aerobic endurance, resistance, and balance exercises for this patient group. For the outcome CIPN, there is less knowledge concerning the effect of exercise, and recently published consensus statements and reviews conclude that the evidence is still insufficient [7, 10, 11].

Before the performance of a full-scale RCT to evaluate the effects of an exercise intervention during adjuvant chemotherapy for CRC, issues of recruitment and retention need to be properly addressed. In addition, exploration of preliminary efficacy (changes) in patient-reported CIPN and fatigue is necessary for the estimation of sample size in the future RCT. On this background, the primary aim of the current study was to evaluate the feasibility of an exercise intervention and data collection among patients during adjuvant treatment for CRC by tracking willingness to participate, inclusion and dropout rate, attendance and adherence to the intervention, safety, and completion rate of questionnaires and physical testing. The secondary aim was to explore post-intervention changes in CIPN and fatigue.

## Material and methods

### Trial design

This was as a single-centre, non-randomized interventional feasibility study with a pre-post design performed at St. Olav’s hospital in Trondheim, Norway. Fourteen months after commencement of the trial, a collaborative hospital (alesund hospital) was invited to participate in the study to prepare this hospital for the future RCT.

### Participants

The eligibility criteria were radical resection for stage II–IV CRC within the last 3 months and scheduled for adjuvant chemotherapy (Resection for synchronous metastases was allowed.), age 18–80 years, performance status 0–2 according to the Eastern Cooperative Oncology Group [12], ability to conduct the intervention based on the treating physician’s assessment, and ability to understand Norwegian language. The exclusion criteria were serious comorbidity contraindicating physical exercise and treatment for other cancers during the 5 past years, except for basal cell carcinoma of the skin and cervical carcinoma in situ.

During the recruitment period, the consulting oncologists screened all patients referred to adjuvant chemotherapy after surgery for CRC for eligibility. The treating oncologist provided oral and written information at the first consultation, and a study coordinator obtained written informed consent within a few days.

### Intervention

The intervention was an individually tailored and supervised exercise programme including progressive aerobic endurance, resistance, and balance exercises. A physiotherapist, certified in giving exercise for cancer patients, supervised the exercise sessions twice a week at a specialized outpatient training facility for cancer patients located within the hospital area. In addition, the participants were encouraged to perform one weekly, unsupervised exercise session with endurance and balance exercises in their home setting. The exercise intervention lasted throughout the period of adjuvant treatment.

Each exercise session consisted of 10-min warm-up, 20-min aerobic endurance, 15-min resistance, and 15-min balance exercises. Participants performed the warm-up and endurance exercise on a treadmill. Endurance exercise was standardized as a gradual approach to intervals of 4 min (Table 1). The Borg’s scale [13] was used to instruct the participants regarding intensity of the endurance exercise and to map the participants’ rate of perceived exertion (RPE). The physiotherapist recorded RPE after warm-up and following each interval. On a scale from 6 (no effort) to 20 (maximal effort), the participants reported how strenuous the exercise was (RPE). For progression, the intensity of the interval training was increased during the intervention period; from 12–14 (‘somewhat hard’) on Borg’s scale in weeks 1–16 to 14–16 (‘hard’) from week 17.

**Table 1:** Endurance and resistance exercise

Aerobic endurance exercise		
Period/exercise	Duration	Borg’s scale
Week 1–2		
Walking on treadmill^1^	1 × 5 min	12–14
Week 3–8		
Intervals of uphill walking	4–6 × 2 min	12–14
Week 9–16		
Intervals of uphill walking	3–4 × 3 min	12–14
Week 17–24		
Intervals of uphill walking	4 × 3–4 min	14–16
Resistance exercise		
Period/exercise	Period	Repetitions (reps)
Week 1–8 Knee extension Sitting chest press Standing rowing Seat raise	Week 1 Week 2 Week 3–8	1 × 12 reps 2 × 12 reps 3 × 12 reps
Week 9–24 Leg press Oblique seated chest press with manuals Standing rowing Lying on back, one leg alternately lowering	Week 9–16 Week 17–24	3 × 10 reps 3 × 8 RM^2^/4 × 6 RM

^1^Getting accustomed to the treadmill

^2^RM = repetition maximum

The resistance exercises were aimed at large muscle groups and followed a period plan that involved individually tailored progression according to standardized training principles (Table 1). During the first 2 weeks, the focus was adaptation, learning of technique, and intensity management. In weeks 3–8, participants performed the exercises with submaximal intensity (low resistance, up to 12 repetitions in three series) to account for any postoperative limitations (e.g., avoiding high abdominal pressure and pain provocation). In weeks 9–16, exercise load was adjusted based on the weight the participant managed to lift a maximum of 10 times and repeated in three series. In the last period (weeks 17–24), intensity was increased by reducing the number of repetitions (6–8) and increasing the number of series (3–4) to work up to maximum strength. In line with individually adapted progression, manual weights, elastic bands, and various exercise equipment were used.

Balance training consisted of a set of exercises, lasting 15–20 min, to be performed on various surfaces (floor, cushions, or Bosu balls). Individual tailoring was based on the physiotherapist making a selection from a standardized pool of exercises with increasing difficulty from static to dynamic balance, and progress was monitored in the two weekly supervised sessions.

### Outcomes

#### Primary outcomes

The rate of consenting participants among those invited for participation defined the feasibility outcome *willingness to participate*. *Inclusion rate* was defined as the number of included participants among eligible participants identified, and *dropout rate* was defined as the number of participants who withdrew from the study among consenting participants. This latter group was termed ‘dropouts’, and the rest were termed ‘completers’.

*Attendance to supervised exercise* was calculated as the number of performed sessions divided by the number of planned sessions. The physiotherapist registered whether the participant met and why he/she did not meet. *Adherence to supervised exercise* was analysed by comparing the content of each session when a participant met with the exercise programme according to protocol. The physiotherapist registered the duration of the warm-up and the endurance exercise, the number and duration of each interval and intensity, the different resistance exercises and number of repetitions, and whether the participant performed the balance exercise. Looking at each component, adherence to endurance, resistance, and balance exercises was analysed, respectively. *Attendance to unsupervised exercise* was calculated by dividing the number of performed unsupervised exercise sessions with the number of unsupervised exercise sessions according to protocol, and it was the physiotherapist that registered whether the home training was done.

*Safety*, recorded as all serious adverse events (SAEs), was registered from the participants who started the intervention until 1 month after the end of the intervention. In addition, any adverse event occurring *during* supervised exercise was noted.

The feasibility of the data collection was measured by the *completion rate of questionnaires and physical testing*. The participants filled in questionnaires at baseline, after 3, 6, 9, and 12 months, and they performed the physical tests at baseline, after 3 and after 6 months. The questionnaires used were The European Organization for Research and Treatment of Cancer Quality of Life Questionnaire C30 (EORTC QLQ-C30) [14], EORTC QLQ—Chemotherapy-Induced Peripheral Neuropathy 20 (CIPN20) [15], and The Fatigue Questionnaire (FQ) [16]. Physical tests were ‘Modified Shuttle walk’, ‘Sit-to-stand’, ‘Tandem stance’, and ‘Unipedal stance’ [17–20]. Demographic variables, clinical characteristics, patient-reported physical activity, and sick leave were also assessed.

#### Secondary outcomes

Secondary outcomes were changes in patient-reported CIPN and fatigue between baseline (T_0_) and 3 months after inclusion (T_1_). CIPN was assessed by the 9-item EORTC QLQ-CIPN20 sensory subscale [15]. Each item is rated on a scale from 1 (‘not at all’) to 4 (‘very much’). Fatigue was assessed by FQ which contains 13 questions. Each question is rated on a scale from 0 (‘not at all’ or ‘less than usual’) to 3 (‘much worse than usual’).

#### Adjuvant chemotherapy and change in assessments

According to the national guidelines at the time this study started, adjuvant chemotherapy for CRC should start within 4–8 weeks postoperatively and last for 24 weeks [2]. Younger patients (< 70 years) should receive combination chemotherapy with intravenous (IV) fluorouracil/calcium folinate or oral capecitabine in combination with IV oxaliplatin. The same guidelines recommended monotherapy with capecitabine or IV fluorouracil/calcium folinate to the elderly patients (> 70 years) [2]. After commencing this study, new recommendations regarding duration of adjuvant chemotherapy was published [21]. As a result, some participants received 12, not 24 weeks of adjuvant treatment. These participants performed physical tests at baseline and after 3 months.

### Sample size

It was estimated that 20 participants could be recruited within a year at St. Olav’s hospital, and this number was considered to be sufficient in evaluating whether the intervention and test procedures were feasible and in estimating the sample size for the larger randomized trial.

### Analytical methods

To estimate adherence to supervised endurance exercise, the total number of minutes of warm-up plus intervals performed for every session was divided by the minimum number of minutes of warm-up and intervals according to the protocol. Similarly, adherence to supervised resistance exercise was estimated by looking at the number of resistance exercises and repetitions performed for every session compared with the protocol. Adherence to supervised balance exercise was estimated by dividing the number of performed supervised balance training by the number of performed supervised sessions.

The raw score (*RS*) in CIPN was calculated by the sum of each item’s score (1–4) divided by the number of items. *RS* = (*I*_1_ + *I*_2_ + … + *In*)/*n*. A linear transformation of the *RS* to 0–100 gives the score (*S*), where higher *S* indicates worse CIPN. *S* = ((*RS*−1)/3) × 100 [22]. For each participant, *S* at *T*_0_ is subtracted from *S* at *T*_1_ to calculate the change in CIPN.

FQ measures physical fatigue (PF) (scores 0–21) and mental fatigue (MF) (scores 0–12). Higher score indicates more fatigue [16]. For each participant, PF and MF scores at *T*_0_ are subtracted from PF and MF scores at *T*_1_ to calculate the changes in PF and MF.

Continuous variables are reported by median values, range, and standard deviation (SD). The statistical analyses performed were descriptive statistics using the IBM SPSS Statistics, version 25.

#### Numbers analysed

Exploring attendance and adherence to the intervention and completion rate of physical tests and questionnaires after baseline, only completers were included. All consenting participants were included when analysing completion rates for baseline testing and questionnaires. Only participants who filled in in CIPN20 and FQ at *T*_0_ and *T*_1_ were included in analysing changes in patient-reported CIPN and fatigue.

## Results

### Recruitment

**Fig. 1:**
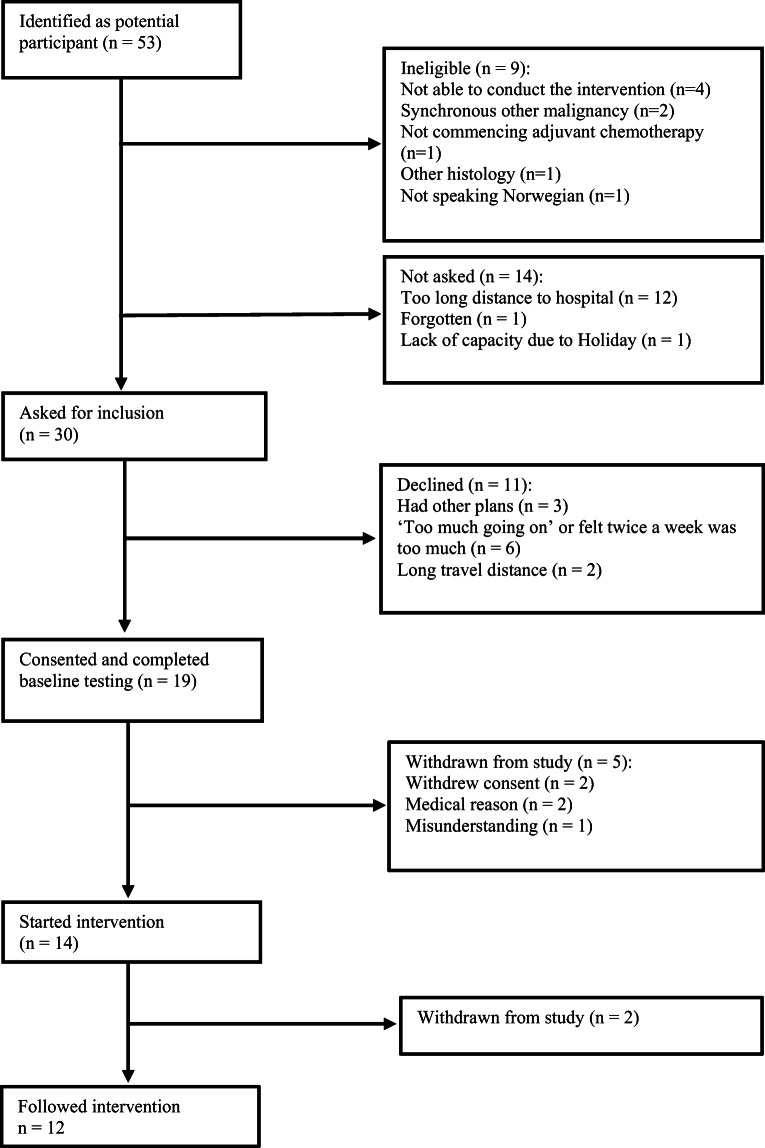
Participant flow

From December 2016 to November 2018, 52 potential participants were identified at the Cancer Clinic, St. Olav’s hospital. One participant was identified and recruited from alesund hospital. Nine patients did not fulfil the inclusion criteria for reasons described in Fig. 1. Fourteen patients were identified as eligible, but not asked to participate. The major reason for not asking was long travel distance to the hospital. After including 19 of the planned 20 participants, the study was closed due to a long period of slow recruitment, and the planned RCT was commencing.

### Baseline data

Table 2 presents baseline demographics and clinical characteristics. Participants received adjuvant chemotherapy for a period of 12 to 24 weeks, with median starting 6 weeks after surgery.

**Table 2:** Baseline demographics and clinical characteristics for completers and the dropouts

	Completers	Dropouts
No. of patients	12	7
Age, years, median [range]	57.5 [33, 78]	69 [43, 80]
Males	7	3
Females	5	4
ECOG PS		
0	7	2
1	4	5
2	1	0
Comorbidity (Charlson comorbidity)		
None	9	5
Cerebrovascular disease (prior TIA or stroke)	2	1
Prior peptic ulcer	1	0
Connective tissue disease	0	1
Stoma		
Yes	0	2
No	12	5
Type of surgery		
Laparoscopy	8	2
Open	4	5
Stage		
III	10	5
IV	2	2
Adjuvant treatment planned		
Combination chemotherapy	11	4
Monotherapy	1	3
Time from surgery to start chemotherapy, days, median [range]	42 [32, 58]	45 [36, 57]
Marital status		
Living alone	3	5
Married/partner	9	2
Employment		
Working	9	1
Partly working/partly disabled	0	1
Retired	3	5
Education		
Elementary or high school	2	6
College/university	10	1

The completers had a lower median age than the dropouts (58 vs. 69 years). A higher proportion of the completers were married or had a partner (9 of 12 vs. 2 of 7) and had higher education than the dropouts (10 of 12 vs. 1 of 7).

### Outcomes

#### Willingness to participate and inclusion and dropout rates

Nineteen among the 30 eligible participants that were invited to take part consented, giving a willingness to participate of 63%. Figure 1 lists reasons for declining participation. With 19 included among 44 eligible participants, the inclusion rate was 43%. Five of the 19 participants never started the intervention. Two participants were hospitalized shortly after the first course of chemotherapy with serious complications, and further adjuvant chemotherapy was stopped. Two participants withdrew consent shortly after inclusion, reporting having ‘too much going’ and having transportation issues, respectively. The fifth dropout was not contacted. Two of 14 participants dropped out after one and four exercise sessions, respectively. One reported pre-existing back pain got worse, and the other did not show up after the first session despite repeated proposals of new appointments. Total dropout rate was 37% (7 of 19).

#### Attendance and adherence to the intervention

Table 3 summarizes attendance and adherence to the supervised exercise. The median rate of attendance to supervised exercise was 85%. Attendance rate was above 77% in 10 of 12 completers. For the two remaining participants, the rate was 33% and 54%, respectively. Reasons for not meeting to a session were that the participant was not feeling well (33%), being hospitalized (15%), being out of town (8%), and other reasons (4%). In 40% of the cases, the reason was unknown, and the participant with the lowest rate of performed sessions accounted for two-thirds of these cases. The median adherence to supervised endurance, resistance, and balance exercises was 96, 95, and 100%, respectively.

**Table 3:** Attendance and adherence to supervised exercise

	According to protocol	*N*	Median	Range	SD
Planned sessions (number)	48	12	44	[22, 46]	7.6
Performed sessions (number)		12	37.5	[12, 46]	11.1
Attendance to supervised exercise (%)		12	85.4	[33.3, 100]	19.9
Adherence to supervised endurance exercise (%)^1^		12	95.8	[81.6, 100]	6.9
Borg’s scale week 1–16	12–14	12	14	[12, 16]	1.1
Borg’s scale week 17–24	14–16	10	13.5	[12, 16]	1.5
Adherence to supervised resistance exercise (%)^1^		12	94.5	[76.5, 100]	6.5
Adherence to supervised balance exercise (%)^1^		12	100	[86.5, 100]	4.3
Did participants achieve 4 times 3–4-min intervals?	*N*				
Yes	4				
No	6				
Not applicable^2^	2				

^1^Adherence to the exercise programme when a participant met

^2^Adjuvant chemotherapy and the intervention lasted less than 17 weeks

The intensity of the endurance exercise was slightly lower in the second period (week 17–24) with a median of 14 in the first (week 1–16), and a median of 13.5 in the second period. Only four participants achieved intervals of 4 times 3–4 min.

Attendance to the unsupervised exercise was systematically registered only in the second half of the completers. Median attendance rate to unsupervised exercise among these six participants was 59% (41.7–87.5).

#### Safety

No adverse events were registered *during* supervised exercise sessions. Two thromboembolic events occurred, where one was a deep vein thrombosis of the lower leg shortly after hospitalization due to an infection. The participant had not been to any supervised exercise the past 10 days before this incident. The other was an incident of pulmonary embolism. The participant received combination chemotherapy 6 days before the first symptoms of pulmonary embolism and did the last supervised exercise 10 days before diagnosis. Both participants were successfully treated ambulatory with anticoagulation and resumed exercise.

Six of 14 participants had one or two admissions to hospital. There were four admissions due to infection, with one due to neutropenic fever. Two admissions were because of chemotherapy-induced enterocolitis, one was with generalized cramps after administration of chemotherapy, and one was because of painful and disabling cramps of the legs after administration of oxaliplatin.

#### Completion rate of questionnaires and physical testing

All 19 participants completed the physical tests according to protocol at baseline. Eighteen of 19 completed the baseline questionnaires, in which one was filled in 2 days after commencing chemotherapy. At 3, 6, and 12 months, all 12 completers returned the questionnaires, with the QLQ-C30 missing in one participant at 12 months. At 9 months, 11 of 12 were completed, with the CIPN20 and FQ missing in one participant. The 12 completers performed all physical tests.

#### Changes in patient-reported CIPN and fatigue

Table 4 reports changes in CIPN, PF, and MF from *T*_0_ to *T*_1_. The symptoms of CIPN increased from *T*_0_ to *T*_1_ with a median increase of 14.8 on a scale from 0 to 100. PF decreased one point on a scale from 0 to 21, and MF increased one point on a scale from 0 to 12.

**Table 4:** Individual changes in patient-reported chemotherapy-induced peripheral neuropathy and fatigue

		CIPN^1^			PF^2^			MF^3^		
	*N*	Median	Range	SD	Median	Range	SD	Median	Range	SD
*T*_0_^4^	10	0.5	[0, 33.3]	10.3	16.0	[6.0, 24.0]	6.3	4.5	[4.0, 8.0]	1.3
*T*_1_^5^	10	20.4	[0, 44.4]	13.0	15.0	[7.0, 25.0]	5.5	5.5	[4.0, 10.0]	2.1
*T*_1_–*T*_0_	10	14.8	[-3.7, 25.9]	9.6	− 1.0	[− 6.0, 13.0]	5.9	1.0	[0, 5.0]	1.6

^1^European Organization for Research and Treatment of Cancer Quality of Life Questionnaire–Chemotherapy-Induced Peripheral Neuropathy 20 sensory subscale (score 0–100)

^2^Physical fatigue from Fatigue Questionnaire (score 0–21)

^3^Mental fatigue from Fatigue Questionnaire (score 0–12)

^4^Baseline

^5^After 3 months

## Discussion

This study investigated the feasibility of a combined supervised and home-based exercise intervention in CRC patients receiving adjuvant chemotherapy. We found a high willingness to participate, attendance and adherence to the exercise intervention, and completion rate of study specific tests. A high proportion dropped out before the start of intervention, and a major barrier for inclusion was long travel distance to participate in supervised exercise.

A high fraction (63%) of the patients were willing to participate. This is higher than in similar studies which have reported willingness to participate between 37% and 49% [23–26]. One possible reason for the high willingness could be the non-randomized design, where all participants could take part in physical exercise. Also the fact that the treating oncologists providing information had a positive attitude towards the study may have contributed to the high willingness. Contrary to our findings, Waart et al. reported difficulties in recruiting patients with colon cancer to an exercise study during adjuvant chemotherapy, experiencing that the clinicians were hesitant to refer patients [26].

Despite the high willingness demonstrated, the inclusion rate was only 43% among eligible patients. Long travel distance was a major barrier, as it made oncologists not asking for participation and patients to decline recruitment. In retrospect, long travel distance should have deemed a potential participant ineligible. However, this was not defined pre-trial, but left to be decided upon by the treating oncologist.

More than one-third of the participants dropped out after inclusion, a higher dropout rate than similar studies, reporting between 6% and 22% [23–25]. However, the majority of the dropouts happened before the start of intervention, mainly due to conditions not controlled by the participants. With the low sample size in this study, small numbers may have large impact on percentage and not necessarily reflecting the expected dropout rate in a larger study.

The attendance and adherence to the supervised exercise were high. A median attendance rate to supervised exercise of 85% is comparable to other studies reporting between 61% and 89% [23, 24, 26]. One likely reason for the high attendance was that the exercise intervention was supervised. Systematic reviews and meta-analyses of RCTs with various cancer types have shown that supervised exercise has a greater effect on several endpoints than unsupervised, and this could be explained by a higher compliance to supervised exercise [27, 28]. When a participant met, adherence to the exercise intervention in our study was close to 100%. A physiotherapist, experienced with patients with cancer, supervised the exercise in a one-to-one manner, and this has likely contributed to the high attendance and adherence.

According to protocol, the intensity of the aerobic endurance exercise should gradually increase during the intervention period. This seemed not feasible as the participants reported slightly lower RPE during the last intervention period (week 17–24). The goal of achieving intervals of 4 times 3–4 min was only reached in one-third of the participants. During the course of adjuvant chemotherapy, patients will typically experience increased fatigue and decreased cardiorespiratory fitness [29, 30]. According to the experience of the present feasibility study, we believe that interval training with increasing intensity is not feasible for the majority of the patients during adjuvant treatment.

One limitation of the present study is the lack of systematically reporting of the unsupervised exercise. Based on the available data, compliance to the unsupervised exercise could be interpreted as lower than the supervised. In a future RCT, a self-reported activity diary will be preferred for documentation of unsupervised exercise. Another limitation is the non-randomized design. We do not know if a randomized design would reduce the willingness to participate. The participants in this study were a selective group willing to attend the exercise intervention. It is reasonable to believe that those willing to participate had a more positive attitude towards exercise than those declining, like Waaet et al. found in their study [26]. Strategies to improve recruitment to interventional studies are needed, and this study did not address that. Because of the higher dropout rate than anticipated, a larger sample size could have strengthened the study. Regarding data collection, we have demonstrated that this was feasible with nearly 100% completion rates of both the physical tests and questionnaires.

There was no temporal relationship between the SAEs and the exercise intervention, and it is most likely that the SAEs reported were related to the chemotherapy, although this needs to be confirmed in an RCT. There were two (14%) thromboembolic events among the 14 participants. In comparison, an adjuvant study comparing two different chemotherapy regimens in CRC reported an incidence rate of thromboembolism of around 6% [6]. With the small sample size in our study, a higher rate of thromboembolism might just be by chance, and no conclusion can be drawn.

As expected, we found that symptoms of CIPN increased from baseline to 3 months after inclusion, as we do not expect exercise to fully prevent development of CIPN. It remains to be established in an RCT if the degree of CIPN developed can be reduced among those randomized to an exercise intervention compared with a control group. Zimmer et al. found that worsening of CIPN could be prevented among metastatic CRC patients receiving palliative chemotherapy randomized to a multimodal exercise programme in a small RCT [31].

To conclude, this study has demonstrated that a combination of supervised and home-based aerobic endurance, resistance, and balance exercises in CRC patients receiving adjuvant chemotherapy was feasible and safe, with the exception of a planned increased intensity of the aerobic endurance exercise which was not feasible for the majority. Based on our experiences from this feasibility study, we have made some adjustments in the ongoing RCT regarding the intervention and data collection, including physiotherapists supervise participants in their local community close to their homes [32], and the endurance exercise is kept on a moderate intensity and with a duration according to general recommendations [33].

## Data Availability

Data can be provided at request.

## Appendix. Illustrations of balance and resistance exercises, with permission from ExorLive

Balanseprogram nivå 1

Balanseprogram nivå 2

Balanseprogram nivå 3

Balanseprogram nivå 4

Styrkeøvelser - Periode 1

Styrkeøvelser - Periode 2
